# Molecular Effects of Cornelian Cherry Fruit (*Cornus mas* L.) Extract on Sleep Deprivation-Induced Oxidative Stress, Cytokine Dysregulation, and Behavioural Changes in Wistar Rats

**DOI:** 10.3390/cimb47060399

**Published:** 2025-05-28

**Authors:** Vlad Sever Neculicioiu, Ioana Colosi, Alexandra Sevastre-Berghian, Dan Alexandru Toc, Horațiu Alexandru Colosi, Luminita David, Mara Muntean, Remus Moldovan, Ana-Maria Vlase, Vlad Alexandru Toma, Carmen Costache, Şoimiţa Mihaela Suciu, Simona Clichici

**Affiliations:** 1 Department of Microbiology, “Iuliu Hatieganu” University of Medicine and Pharmacy, 400349 Cluj-Napoca, Romania; 2 Department of Physiology, “Iuliu Hatieganu” University of Medicine and Pharmacy, 400006 Cluj-Napoca, Romania; 3 Division of Medical Informatics and Biostatistics, Department of Medical Education, “Iuliu Hatieganu” University of Medicine and Pharmacy, 400349 Cluj-Napoca, Romania; 4 Research Centre for Advanced Chemical Analysis, Instrumentation and Chemometrics, Faculty of Chemistry and Chemical Engineering, Babes-Bolyai University, 400347 Cluj-Napoca, Romania; 5 Department of Cell and Molecular Biology, Faculty of Medicine, “Iuliu Hațieganu” University of Medicine and Pharmacy, 400349 Cluj-Napoca, Romania; 6 Department of Pharmaceutical Botany, Faculty of Pharmacy, “Iuliu Hațieganu” University of Medicine and Pharmacy, 8 Victor Babeș Street, 400012 Cluj-Napoca, Romania; 7 Department of Molecular Biology and Biotechnology, Faculty of Biology and Geology, Babeș-Bolyai University, 44 Republicii Street, 400015 Cluj-Napoca, Romania

**Keywords:** REM sleep, PSD, antioxidants, brain, hippocampus, cortex, tumour necrosis factor-alpha, Interleukin-6, electron microscopy

## Abstract

Sleep deprivation (SD) induces significant neurobiological changes, including oxidative stress, neuroinflammation, and behavioural impairments. This study was designed as a proof of concept to assess the potential for modulating the effects of SD through a short-term seven-day administration of *Cornus mas* (*C. mas*) in a rapid eye movement (REM) SD rodent paradigm. Adult male Wistar rats were randomised in four groups (n = 7): control, *C. mas* (CM), sleep deprivation (SD), and sleep deprivation with *C. mas* (SD + CM). Behaviourally, SD induced hyperactivity and hyperlocomotion. SD determined histological alterations in the prefrontal cortex and corpus callosum myelin coupled with ultrastructural mitochondrial and cellular abnormalities in the prefrontal cortex, hippocampus, and pineal gland. Despite evidence of systemic oxidative stress coupled with decreased serum GABA and BDNF following SD, no significant changes were observed in redox markers or inflammatory cytokine levels (TNF-α, IL-1β) within the prefrontal cortex or hippocampus. *C. mas* extract has shown an overall modest modulatory action, mainly evidenced on behavioural, histological, and ultrastructural parameters. Taken together, these findings highlight behavioural changes and region-specific molecular and structural abnormalities following prolonged REM SD in rats.

## 1. Introduction

Sleep loss, whether stemming from sleep deprivation (SD) or sleep restriction (SR), appears to be a defining characteristic of the contemporary 24/7 technological world, due to occupational or personal constraints and obligations. Multiple datapoints seem to indicate that approximately one-third of the adult population [1,2] and more than two-thirds of the young segment of the population are sleep deprived [3,4]. In humans, both acute SD and chronic SR have been shown to elicit a wide range of neuro-behavioural deficits [5]. Furthermore, prolonged sleep curtailment has been associated in multiple prospective studies with a wide range of negative health outcomes such as cardiovascular, metabolic, and increase in all-cause mortality [6]. The link between sleep curtailment and adverse health outcomes is not fully understood, likely involving multiple mechanisms. Oxidative stress (OS) and inflammation have been proposed as key factors connecting disrupted sleep to cardiovascular, metabolic, and neurodegenerative disorders (reviewed in [7,8,9]).

Recent data in *Drosophila* seem to point towards a bidirectional relationship between sleep and OS [10]. This role of sleep is further supported by experimental rodent data. Most available evidence indicates that both paradoxical (PSD) and total sleep deprivation (TSD) induce OS in central and peripheral sites [11,12]. Furthermore, OS during SD can influence multiple response pathways such as a disruption in oxidative phosphorylation and decoupling of ATP synthesis, induction in the endoplasmic reticulum unfolded protein response (UPR), and ultimately mitochondrial dysfunction (reviewed in [13]).

Interest for herbal medicines and products has increased in recent years [14], influenced among other factors by availability, affordability, applicability, and accessibility [15]. Cornellian cherry (*Cornus mas* L.) is a deciduous shrub from the genus *Cornaceae*, widely distributed in Europe and Asia [16]. *Cornus mas* (*C. mas*) fruits have been traditionally used both as a fresh or processed food source (syrups, jams, alcoholic drinks, etc.) and for their medicinal properties in folk medicine (gastrointestinal, respiratory, urinary disorders, fever, infections, inflammation, and others) [17].

The primary bioactive constituents within *C. mas* fruits, flowers, and leaves are relatively well known and encompass mainly polyphenols (e.g., anthocyanins, flavonoids, flavanols, phenolic acids, tannins), but also iridoids, carotenoids, vitamins, organic and fatty acids. These constituents exhibit a diverse range of in vitro and in vivo pharmacological properties such as antidiabetic, hypolipidemic, hepato-, cardio- and neuroprotective, antioxidant, anti-inflammatory, anticancer, and antibacterial effects (reviewed in [18]). Some of these effects have also been investigated in human clinical studies: anti-obesity and metabolic effects in metabolic dysfunction-associated fatty liver disease (MAFLD) [19], anti-hypertensive in MAFLD [20], menopausal-related bone health [21], possible improvements in the lipid profile and vascular inflammation in children with dyslipidaemia [22], suggesting translational health benefits of different types of *C. mas* formulations. In addition to the effect of each constituent, it is well known that whole plant extracts may present different and augmented effects based on the synergy or polyvalent action between constituents [23,24]. Collectively, the health benefits attributed to polyphenols are more likely the result of combined actions of various phytochemicals, rather than the effect of a single compound [25].

Various extracts of *C. mas* fruits have demonstrated antioxidant and anti-inflammatory effects in several short-term treatment regimens in rodents ranging from one to seven days [26,27,28,29]. However, neuroprotective effects have primarily been explored in studies involving prolonged administration over several weeks, specifically with the dietary inclusion of cornelian cherries [30] and flavonoids derived from *C. mas* [31].

This study was designed as proof of concept to evaluate the modulatory potential on the effects of SD with a short-term administration of cornelian cherry extract, based mainly on its proposed antioxidant and anti-inflammatory activities. To the best of our knowledge, this report is the first to evaluate the effects of a *C. mas* fruit extract in a model of PSD on Wistar rats.

## 2. Materials and Methods

### 2.1. Cornelian Cherry Fruit Extract: Preparation, Characterisation, and In Vitro Antioxidant Activity

#### 2.1.1. Fruit Extract Preparation

Fresh cornelian cherry fruits (*C. mas*) were purchased from a local market in Cluj-Napoca in June 2023. The fruits were washed for impurities with running and distilled water, dried, and immediately frozen until further use. The fruits were identified by Assistant Professor Ana-Maria Vlase, PhD, and previously registered with the Botany Department of UMF “Iuliu Hatieganu” Cluj-Napoca, Romania with the Herbarium voucher code UMF Cluj-Napoca 66.1.1.1/06.2023.

The fruit extract was obtained through the following procedure: cherry fruits were destoned and 200 g mashed fruit paste was mixed with 500 mL acetone (mass ratio 1:2.5); the mixture was kept in a magnetic stirrer at room temperature for four hours, filtered and vacuum concentrated at 40 °C until acetone was completely eliminated.

#### 2.1.2. Total Polyphenol Content Evaluation

The total polyphenol content was assessed using the Folin–Ciocalteu method [32], with slight modifications as previously outlined [33]: 1.5 mL of Folin–Ciocalteu reagent was combined with 0.25 mL of fruit extract, and the mixture was incubated for 5 min and mixed with 1.2 mL of a 0.7 M sodium carbonate solution. Subsequently, the mixture was stored in a dark environment at room temperature for two hours. The absorbance was determined at 765 nm in a Lambda 25 UV–Vis spectrophotometer (Perkin-Elmer, Shelton, CT, USA). The result was determined through a calibration curve established for gallic acid and expressed in grams of gallic acid equivalents (GAE)/1000 mL of extract.

#### 2.1.3. Liquid Chromatography Tandem Mass Spectrometry Analysis (LC-MS/MS)

The phytochemical composition of *C. mas* fruit extract was evaluated using LC-MS/MS employing two previously validated analytical methodologies [34,35,36]. The equipment utilised included the Agilent Technologies 1100 HPLC Series system (Agilent, Santa Clara, CA, USA), equipped with an autosampler, column thermostat, binary gradient pump, degasser, and UV detector. This system was integrated with an Agilent Ion Trap 1100 SL mass spectrometer (LC/MSD Ion Trap VL) [34,35,37].

In the first analytical method, chromatographic separation was performed on a reverse-phase Zorbax SB-C18 analytical column (100 mm × 3.0 mm i.d., 3.5 μm particle size, Agilent Technologies—Agilent, Santa Clara, CA, USA) using a mobile phase of methanol and 0.1% acetic acid (*v*/*v*) in a binary gradient. The elution began with a linear gradient starting from 5% methanol, increasing to 42% over 35 min, followed by isocratic elution at 42% methanol for 3 min, and re-equilibrated with 5% methanol over the next 7 min [36,37]. The column temperature was maintained at 48 °C, with a flow rate of 1 mL/min and an injection volume of 5 μL. Detection was carried out using UV and MS modes, setting the UV detector at 330 nm for polyphenolic acids up to 17 min and at 370 nm for flavonoids and their aglycones up to 38 min. The MS operated in electrospray ionisation (ESI) negative mode, with a capillary voltage of +3000 V, a nebuliser pressure of 60 psi (nitrogen), and a gas flow rate of 12 L/min at 360 °C [34,35,36,37].

A second validated LC-MS analytical method was employed to potentially identify six additional polyphenols: epicatechin, catechin, syringic acid, gallic acid, protocatechuic acid, and vanillic acid. The same analytical column and equipment settings were used with a modified mobile phase gradient starting at 3% methanol, increasing to 8% at 3 min and reaching 20% by 8.5 min, maintained up to 10 min before returning to 3%. The analysis of bioactive compounds was performed in MS mode with similar ESI conditions as the first method [38,39].

A new LC-MS method has been developed and validated for the quantification of 13 anthocyanins in *C. mas* fruit extract, namely delphinidin 3-galactosid, delphinidin 3-glucosid, cyanidin-3-O-galactosid, delphinidin 3-rutinosid, cyanidin 3-glucosid, cyanidin 3-arabinosid, cyanidin 3-rutinosid, petunidin 3-glucosid, delphinidin, malvidin 3-glucosid, cyanidin, petunidin, and pelargonidin. For this purpose, the same equipment and analytical column as described in the previous methods were used [39]. Detection of anthocyanins was performed with a DAD detector in the LC-MS system. Separation was carried out using a gradient elution, consisting of acetonitrile and 0.1% trifluoroacetic acid in water (*v*/*v*). Detection was performed in either DAD, MS, or MS/MS mode using positive ionisation with an ion trap mass spectrometer equipped with an ESI source. The DAD detector was set at 530 nm with a bandwidth of 20 nm and a reference wavelength of 700 nm. From the available analytical standards mentioned above, only one compound was identified and quantified in the *C. mas* extract.

The identification of each bioactive compound involved comparison of MS spectra/traces with library standards, followed by quantification using UV detection, considering calibration curves of corresponding analytical standards. The results were processed using DataAnalysis (v5.3) and ChemStation (vB01.03) software from Agilent, with quantifications expressed as micrograms per mL of vegetal extract.

#### 2.1.4. In Vitro Antioxidant Activity—DPPH Radical Scavenging Activity

The antioxidant capacity of the extract was evaluated in vitro using the previously described DPPH (2,2-diphenyl-1-picrylhydrazyl) assay, a method known for its effectiveness in assessing radical scavenging activity [34,40]. Initially, DPPH was dissolved in methanol to a concentration of 1 mg/mL and then diluted to 40 μg/mL for the assay. In the procedure, 200 μL of this DPPH solution was mixed with 50 μL of the extract (diluted 1:200) and incubated for 30 min in the dark. The change in absorbance was measured at 517 nm using a BMG Spectrostar Nano microplate reader (BMG Labtech, Ortenberg, Germany) to determine the reduction in DPPH radicals. Antioxidant activity was quantified based on the difference in absorbance between the control solution and the sample [41]. Calibration curves ranged from 1.22 to 122 μg/mL, with a determination coefficient (R^2^) of 0.995, using Trolox as a reference standard. The experiment was carried out in triplicate and the result was expressed in milligrams of Trolox equivalent (TE) per mL of extract.

### 2.2. Experimental Animals

The study protocol was designed a priori and not previously registered. All animal experimental work was approved by the Ethics Committee of the “Iuliu Hatieganu” University of Medicine and Pharmacy Cluj-Napoca, Romania (no. 370/13 June 2023) and all animal procedures followed and respected the 2010/63/EU Directive and local legislative framework regarding the protection of animals used for scientific purposes.

A total of 28 Wistar albino male rats were used in the experiment (body weight 200–250 g, 12–16 weeks). The animals were provided by the animal facility of the University of Medicine and Pharmacy “Iuliu Hatieganu” Cluj-Napoca, Romania. Upon arrival, the animals were acclimatised for seven days before the start of the experimental period. The animals were housed in groups (3–4 animals/cage), in clear plastic cages (Tecniplast Institute, Bucharest, Romania), with standard aseptic wood chip bedding under standard laboratory conditions: 12 h light–12 h dark cycles (lights on at 7 a.m.), temperature 24 ± 2 °C, and relative humidity 55% ± 5. Standardised normocaloric pellets and drinking water were available ad libitum to all experimental groups during the acclimatisation and experimental periods. Experimental work was carried out during the light phase by the same two trained researchers.

### 2.3. Experimental Design

Wistar male rats are among the most frequently used in SD studies [11]. The animals were randomly divided into four groups (n = 7): Control (Control), *C. mas* (CM), sleep deprivation (SD), and sleep deprivation with *C. mas* (SD + CM). Randomisation was performed with the online GraphPad QuickCalcs randomiser utility available at: https://www.graphpad.com/quickcalcs/randmenu/ (accessed on 30 November 2023). The experimental unit was defined as a single animal.

**Figure 1:**
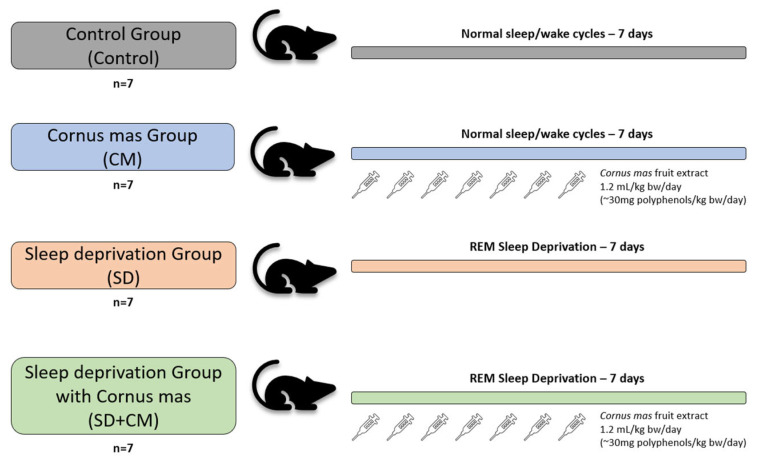
Group formation and treatment summary. A total of 28 Wistar male rats were randomised into four groups (n = 7): control (Control), *C. mas* (CM), sleep deprivation (SD), and sleep deprivation with *C. mas* (SD + CM). The CM and SD + CM groups received *C.mas* fruit extract via oral gavage 1.2 mL/kg bw/day (~30 mg polyphenols/kg bw/day) for seven consecutive days, while control and SD groups received distilled water. The SD and SD + CM groups underwent SD using the modified multiple small platforms (MMSP) protocol for seven consecutive days, while the remaining groups were housed in standardised cages.

The CM and SD + CM groups received *C. mas* fruit extract through oral gavage in a single daily dose of 1.2 mL/kg bw/day (~30 mg polyphenols/kg bw/day) for seven days, during the SD induction. The control and SD groups received a similar volume of distilled water through oral gavage for seven days. The SD and SD + CM groups were exposed to sleep deprivation through the MMSP protocol for seven consecutive days. The control and CM groups were housed in standardised home cages for the duration of the experimental period. The group organisation is summarised in Figure 1. Inclusion criteria were established a priori and consisted of healthy experimentally naive male rodents with similar weight and age. The order of behavioural testing and sample collection was randomised. Multiple outcome measures were assessed: behavioural changes, ultrastructural and histopathological changes, OS parameters, inflammatory markers and serum corticosterone, BDNF, GABA. Outcome assessment and data analysis were performed in a blinded manner.

### 2.4. Sleep Deprivation Induction

The modified multiple platform method (MMSP) was used as previously described [42], with some limited minor modifications. Social hierarchy was maintained through the acclimatisation and experimental period. Animals were kept in socially stable groups of 3–4 rats in large plastic tanks containing small circular platforms (6.5 cm diameter, 7 cm height, 7 cm edge to edge distance between platforms). The size of the platforms was chosen based on previously outlined data [42,43,44,45]. The platform size provided sufficient room for movement, standing, and resting but prevented REM sleep through muscle atonia. The spacing between platforms allowed the animals to freely move between them. The tanks were filled with water of 22–25 °C up to 1–2 cm below the top of the platforms. Upon reaching REM sleep, the animals touched or fell in the water, thus being selectively deprived of paradoxical sleep.

Habituation to the SD tanks was performed prior to the start of the procedure for 1–2 h on three consecutive days (9–11 a.m. daily), for stress reduction and to provide the animals with an opportunity to learn to balance on the platforms. The control and CM groups remained in the standardised cages with sleep ad libitum throughout the experimental period. Food and water were readily available through the experimental period.

**Figure 2:**
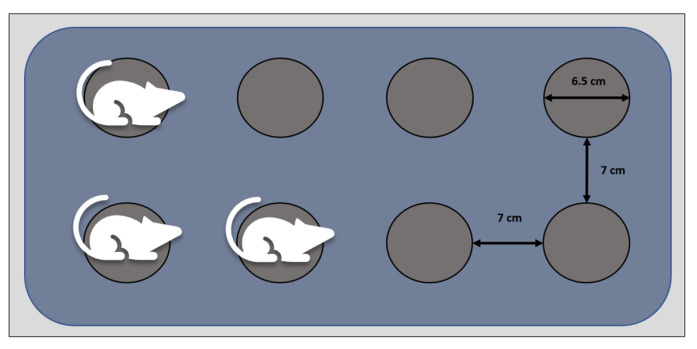
Sleep deprivation (SD) tanks—schematic representation. Socially stable groups of animals were exposed to SD through the modified multiple small platforms (MMSP) protocol for seven consecutive days [42] (65 mm diameter, 70 mm distance between platforms, 70 mm height). Muscle atonia prevented rapid eye movement sleep (REM) and resulted in animals falling or touching the surrounding water. Standard chow and water were available ad libitum.

All animals were manipulated daily by the same two researchers for a limited time between 7 and 9 a.m. to clean the SD tanks and replace the water, change feed and water, and for drug or vehicle administration. The animals were monitored during this timeframe and sleep was prevented by gently tapping the cages. The water was changed daily through the experimental period and the tanks were thoroughly cleaned and disinfected daily with 70% ethyl alcohol. A schematic representation of the SD tanks is presented in Figure 2.

### 2.5. Behavioural Tests—Locomotor and Emotionality-like Behaviour

Two behavioural tests were conducted at the end of the SD paradigm in order to evaluate general locomotion and emotionality-like behaviour: Open Field Test (OFT) and Elevated Plus Maze (EPM) (for each group, n = 6–7) [46,47].

OFT and EPM were used to evaluate general locomotion and emotionality-like behaviour in specific mazes for rats (Ugo Basil Animal Mazes for Video-Tracking, Gemonio, VA, Italy). For each animal, the behaviour was continuously recorded for five minutes through a visual tracking system (Smart Basic Soft-ware version 3.0 Panlab Harvard Apparatus, Barcelona, Spain).

In the OFT, the animals were freely allowed to explore an open field arena (100 × 100 × 40 cm) for 5 min. The total and peripheral travelled distance and number of entries are common measures for general locomotor activity. High centre travelled distance and number of entries and high centre time ratio (centre/total time) are reported parameters of a low level of anxiety. Therefore, measures of both locomotor activity (total travelled distance, travelled distance in periphery, total number of entries, number of entries in periphery) and anxiety-like behaviour (travelled distance in centre, number of entries in centre, time spent in centre/total time) were recorded in the OFT.

While the EPM is considered the gold standard for the evaluation of anxiety in the basic research, it also measures the general locomotor activity. The plus-shaped maze consists of two open (10 × 50 cm) and two closed (10 × 50 × 40 cm) arms that are elevated above the ground level at 60 cm. Total and closed arm travelled distance and number of entries are variables of motor activity in EPM. High open arm travelled distance and number of entries and high open arms time ratio (open arms/total time) indicate low anxiety-like behaviour. Therefore, behavioural parameters for motor activity (total and closed arm travelled distance and number of entries) and for anxiety-related behaviour (open arm travelled distance and number of entries and open arms time ratio (open arms/total time), were recorded in the EPM.

### 2.6. Sample Collection

Upon completion of behavioural tests, blood was collected through retroorbital plexus/sinus sampling under mild anaesthesia (2.5 mg/100 gbw ketamine 10% and 50 mg/100 gbw of xylazine hydroxichloride 2%). The sampled blood was centrifuged for 10 min 6500 rpm and serum was immediately stored at −80 °C until further analysis. Following blood collection, all animals were sacrificed through intraperitoneal administration of ketamine (90 mg/kg body weight) and xylazine (10 mg/kg body weight). The procedure was carried out within the same 3–4 h window during the light phase for all groups, in order to maintain the same circadian window and minimise the circadian influence on further determinations.

**Figure 3:**
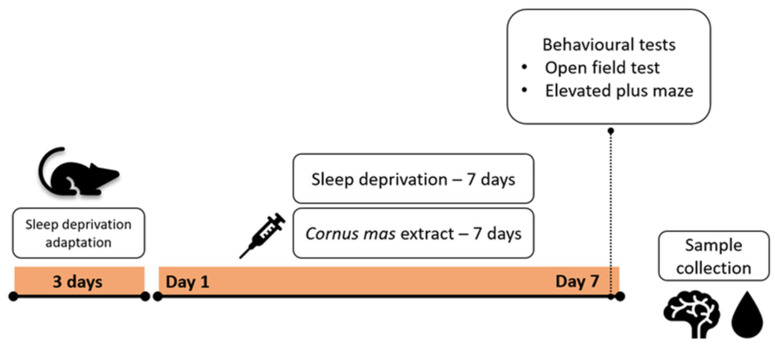
Experimental timeline summary.

Immediately after being sacrificed, the brain was removed and placed on crushed ice. The prefrontal cortex, hippocampus, and pineal gland were identified and dissected. Samples were processed according to the intended determinations as further outlined. The experimental timeline is summarised in Figure 3.

### 2.7. Histopathological Examination

Brain samples were collected for routine Haematoxylin-Eosin (HE) staining (n = 2/group). Brain tissue was fixed in 10% neutral-buffered formalin and embedded in paraffin wax. Paraffin was removed through two successive 5 min washes with xylene and rehydrated through a series of ethanol solutions (100%—two 5-min washes; 95%—5 min, 70%—5 min). Slides were stained with Haematoxylin for 3 min and with eosin for 1 min. Following staining, the tissue sections were dehydrated through a series of ethanol solutions (70%—5 min; 95%—5 min; 100%—two 5 min washes). The slides were then cleared in xylene, mounted and examined in light microscopy in a blinded manner.

### 2.8. Transmission Electron Microscopy (TEM)

Hippocampus, prefrontal cortex, and pineal gland samples were collected for TEM (n = 2/group). After collection, the samples were immediately fixed in a 2.7% glutaraldehyde solution with a 0.1 M phosphate buffer and washed 4 times in the same buffer. Following postfixation with 1.5% osmium tetroxide in 0.15 M phosphate buffer, the samples were dehydrated in acetone solutions of increasing concentrations (30–100%), infiltrated and embedded in EMbed 812 epoxy resin (Electron Microscopy Sciences, Hatfield, PA, USA). Ultrathin sections of 70–80 nm were obtained with a Diatome A382 diamond knife (Diatome, Hatfield, PA, USA), on a Bromma 8800 ULTRATOME III (LKB, Stockholm, Sweden). The sections were collected on 300 mesh copper grids, double-contrasted with uranyl acetate and lead citrate, and examined at 80 kV with a JEOL JEM-100CX II transmission electron microscope (JEOL, Tokyo, Japan). The final images were obtained using a MegaView G3 camera controlled with a Radius 2.1 software, both provided by Emsis (Münster, Germany). The resulting images were interpreted in a blinded manner.

### 2.9. Oxidative Stress Parameters, Inflammatory Cytokines and Corticosterone, BDNF, GABA

OS parameters (reduced Glutathione—GSH, oxidised Glutathione—GSSG, GSH/GSSG ratio, Malondialdehyde—MDA) were determined from prefrontal cortex, hippocampus (n = 5/group) and serum samples (n = 6–7/group). Two cytokines with mainly inflammatory roles (TNF-α, IL-1β) were determined from prefrontal cortex and hippocampus samples (n = 5/group). Furthermore, Corticosterone (CORT), brain-derived neurotrophic factor (BDNF), and Gamma-aminobutyric acid (GABA) were determined from serum samples (n = 6–7).

Brain tissues were homogenised on ice (POLYTRON PT 1200 E, Kinematica, Malters, Switzerland) in 50 mM TRIS and 10 mM EDTA buffer at pH 7.5. Supernatant was collected after centrifugation (1000× *g*, 10 min, 4 °C), stored in pre-labelled sterile Eppendorf tubes, transported on crushed ice and immediately frozen at −80 °C until further analysis. Sample protein content was determined through the Bradford method [48].

MDA levels were measured fluorometrically as the final products of polyunsaturated fatty acids peroxidation, following the method previously described by Conti et al. [49]. Tissue homogenates and serum were incubated in a boiling water bath for one hour in a solution containing 10 mM 2-thiobarbituric acid in 75 mM K_2_HPO_4_ buffer (pH 3.0). Following n-butanol extraction, MDA content was determined spectrofluorometrically (excitation—534 nm; emission 548 nm; Perkin Elmer, Shelton, CT, USA) and expressed as nmol/mL and nmol/mg protein.

GSH and GSSG levels were determined as previously described [50]. Supernatant was diluted with 1.8 mL phosphate buffer (0.1 M, pH 8, containing 5 nmol/L EDTA) and 0.1 mL of o-Phthalaldehyde diluted in methanol (1 mg/mL). Fluorescence was measured after a 15 min incubation window (excitation—350 nm, emission—420 nm) and GSH concentration was determined through a standard curve, expressed as nmol/mL and nmol/mg protein. To determine GSSG levels, 250 µL of supernatant was incubated with N-ethylmaleimide (40 nmol/L) for 30 min, followed by the addition of 0.65 mL of NaOH 0.1 N. The sample was then processed similarly to GSH, with the phosphate buffer replaced by 0.1 mL of a mixture containing 1.8 mL of 0.1 N NaOH and 0.1 mL of o-Phthalaldehyde solution. In addition, the GSH/GSSG ratio was determined as a widely used marker of OS.

TNF-α and IL-1β in brain samples were determined through Rat ELISA kits, as per the manufacturer’s instructions (Elabscience, Houston, TX, USA), with absorbance readings at 450 nm. CORT, BDNF, and GABA in serum samples were determined through Rat-specific ELISA kits following manufacturer’s instructions (CORT and BDNF—Elabscience, Houston, USA; GABA—Fine Biotech, Wuhan, China). The results were expressed as unit OD/mg protein (TNF-α and IL-1β) and pg/mL or ng/mL (CORT, BDNF, GABA) with the associated standard deviations.

### 2.10. Statistical Analysis

All obtained values were collected in Microsoft Excel 2021 (Microsoft, Redmond, WA, USA). The values were analysed and graphed with GraphPad Prism version 9.0 for Microsoft Windows (GraphPad Software, San Diego, CA, USA—https://www.graphpad.com/). Data were analysed by two-way ANOVA to assess the main effects of sleep and treatment, as well their interaction. Significant effects were further analysed through Tukey’s post hoc test. Data were expressed as the Mean ± SD, unless otherwise stated. A *p*-value < 0.05 was considered statistically significant.

## 3. Results

### 3.1. Cornelian Cherry Fruit Extract Characterisation

The total polyphenolic content and in vitro antioxidant activity results of the cherry extract are presented in Table 1.

**Figure 4:**
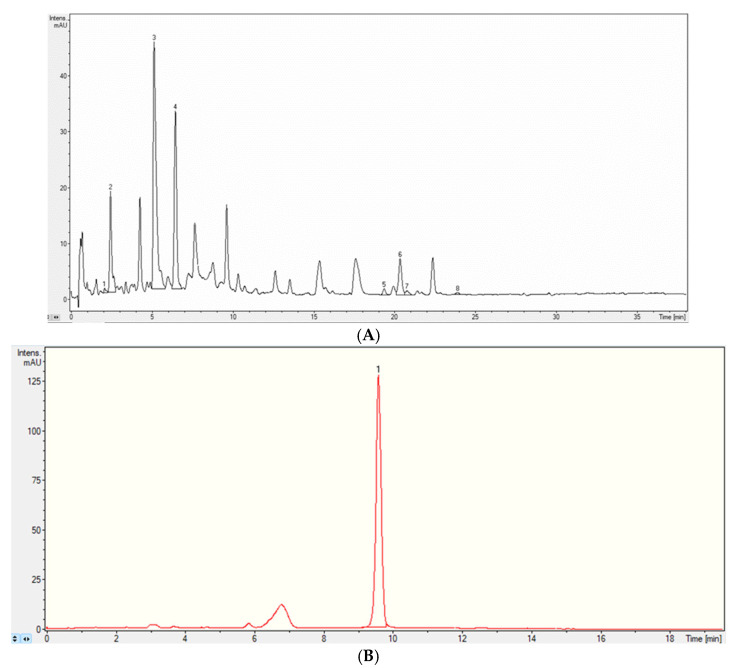
(**A**) Liquid chromatography tandem mass spectrometry (LC-MS) UV chromatogram of polyphenols in cornelian cherry (*C. mas*) fruits extract—first analytical method. Identified compounds: (1) Caftaric acid, (2) Gentisic acid, (3) Caffeic acid, (4) Chlorogenic acid, (5) Hyperoside, (6) Isoquercitrin, (7) Rutoside, (8) Quercitrin. (**B**) Liquid chromatography tandem mass spectrometry (LC-MS) UV chromatogram of anthocyanins in cornelian cherry (*C. mas*) fruits extract. Identified compounds: (1) cyanidin 3-glucoside.

The LC-MS analysis revealed the presence of 14 phenolic compounds. The analysis identified six phenolic acids (caftaric acid, gentisic acid, caffeic acid, chlorogenic acid, gallic acid, protocatechuic acid) and eight flavonoids (hyperoside, isoquercitrin, rutoside, quercitrin, epicatechin, catechin, epigallocatechin, epigallocatechin gallate). The major identified compounds were caffeic acid, chlorogenic acid, gallic acid, isoquercitrin and caftaric acid; one anthocyanin was identified through LC-MS, namely cyanidin-3-glucoside in high amounts (Figure 4A,B and Table 2).

### 3.2. Behavioural Tests

#### 3.2.1. Locomotor Activity

**Figure 5:**
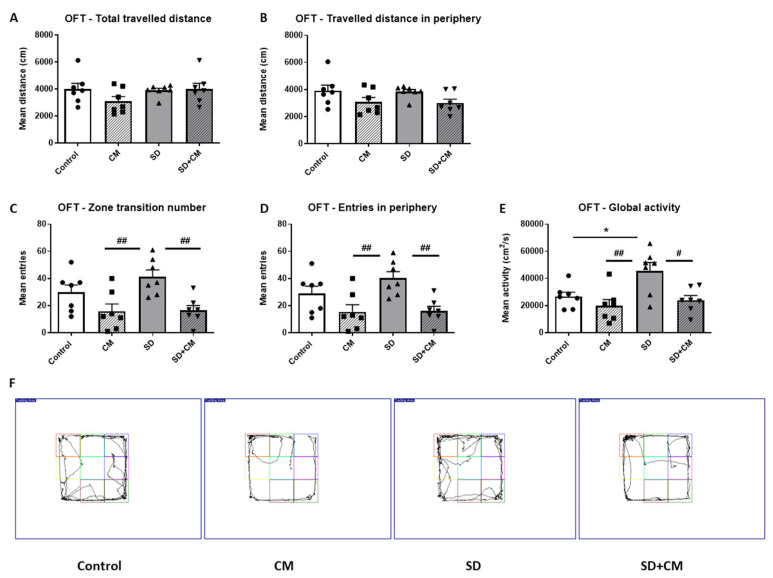
The effects *C. mas* administration on general locomotion in the Open Field Test (**A**–**F**): total (**A**) and peripheral (**B**) travelled distance, total (**C**) and peripheral (**D**) number of entries and global activity (**E**), tracking plots (**F**). Control—control group; CM—*C. mas* group; SD—sleep deprivation group; SD + CM—sleep deprivation with *C. mas* group. Results are expressed as mean ± SEM (n = 6–7). * indicates a significant difference at *p* < 0.05 when compared to the control group; #, ## indicate significant differences at *p* < 0.05 and *p* < 0.01 when compared to the SD group.

The effects of *C. mas* administration on general locomotion in the OFT (Open Field Test) are illustrated in Figure 5. In the OFT, two-way ANOVA revealed a main effect of treatment in the case of distance travelled in the periphery (F = 6.86, η^2^ = 0.22, *p* < 0.05), zone transition (F = 15.55, η^2^ = 0.37, *p* < 0.001), entries in the periphery (F = 15.17, η^2^ = 0.36, *p* < 0.001), and a main effect of treatment and sleep in the case of global activity (F = 9.93, η^2^ = 0.23, *p* < 0.01 and F = 6.59, η^2^ = 0.15, *p* < 0.05). No significant interaction effect between treatment and sleep was observed. In the OFT, the SD group exhibited a significantly higher global activity as compared to the control group (Figure 5E, *p* < 0.05). The CM group exhibited a significantly decreased locomotor activity (zone transition number (Figure 5C), entries in periphery (Figure 5D), and global activity (Figure 5E), *p* < 0.01) in comparison to the SD group. Furthermore, the SD + CM group made fewer zone transitions (Figure 5C, *p* < 0.01), entries in the periphery (Figure 5D, *p* < 0.01), and had a significantly decreased global activity (Figure 5E, *p* < 0.05) compared to the SD group. The CM group tended to travel less (Figure 5) compared to the control group, but the differences were not statistically significant (*p* > 0.05).

**Figure 6:**
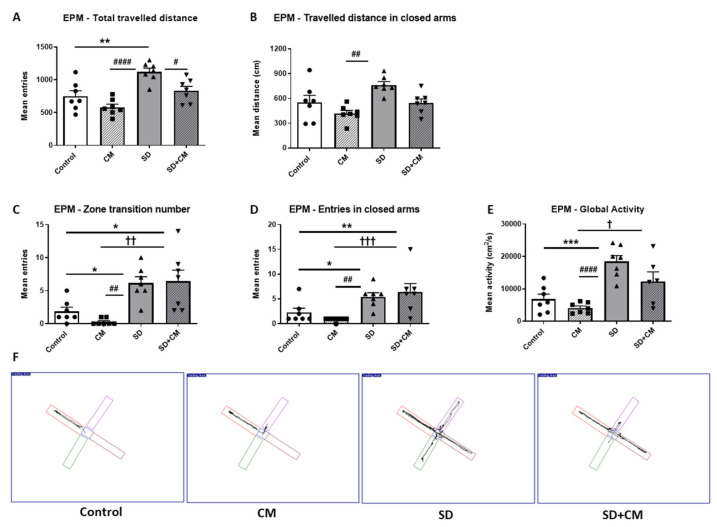
The effects of *C. mas* administration on general locomotion in the Elevated Plus Maze (**A**–**F**): total (**A**) and closed arms (**B**) travelled distance, total (**C**) and closed arms (**D**) number of entries and global activity (**E**), tracking plots (**F**). Control—control group; CM—*C. mas* group; SD—sleep deprivation group; SD + CM—sleep deprivation with *C. mas* group; Results are expressed as mean ± SEM (n = 6–7). *, **, *** indicate significant differences at *p* < 0.05, *p* < 0.01, and *p* < 0.001 when compared to the control group; #, ##, #### indicate significant differences at *p* < 0.05, *p* < 0.01 and *p* < 0.0001 when compared to the SD group; †, ††, ††† indicate significant differences at *p* < 0.05, *p* < 0.01 and *p* < 0.001 when compared to the CM group.

The effects of CM administration on general locomotion in the Elevated Plus Maze (EPM) are illustrated in Figure 6. Two-way ANOVA revealed a significant main effect of treatment and sleep in regard to total travelled distance (F = 12.50, η^2^ = 0.21, *p* < 0.01 and F = 23.25, η^2^ = 0.38, *p* < 0.0001), distance travelled in closed arms (F = 9.76, η^2^ = 0.22, *p* < 0.01 and F = 9.13, η^2^ = 0.21, *p* < 0.01), global activity (F = 5.92, η^2^ = 0.09, *p* < 0.05 and F = 29.72, η^2^ = 0.49, *p* < 0.0001), and a significant main effect of sleep in the case of zone transition number (F = 26.12, η^2^ = 0.51, *p* < 0.0001) and number of entries in closed arms (F = 18.36, η^2^ = 0.42, *p* < 0.001). No significant interaction effect between treatment and sleep was observed. The total travelled distance (Figure 6A, *p* < 0.01), the zone transition number (Figure 6C, *p* < 0.05), the entries in the closed arms (Figure 6D, *p* < 0.05), and the global activity (Figure 6E, *p* < 0.001) were significantly increased in the SD group as compared to the control group. The CM-treated rats travelled less (Figure 6A, *p* < 0.0001 and Figure 6B, *p* < 0.01), made fewer transitions and entries in the closed arms (Figure 6C, *p* < 0.01 and Figure 6D, *p* < 0.01), and were less active (Figure 6E, *p* < 0.0001) as compared to the SD group. Compared to the SD group, the SD + CM group travelled significantly less (Figure 6A, *p* < 0.05) and presented a tendency towards a decreased distance travelled in closed arms (Figure 6B, *p* > 0.05) and lower activity (Figure 6E, *p* > 0.05).

#### 3.2.2. Emotionality-like Behaviour

**Figure 7:**
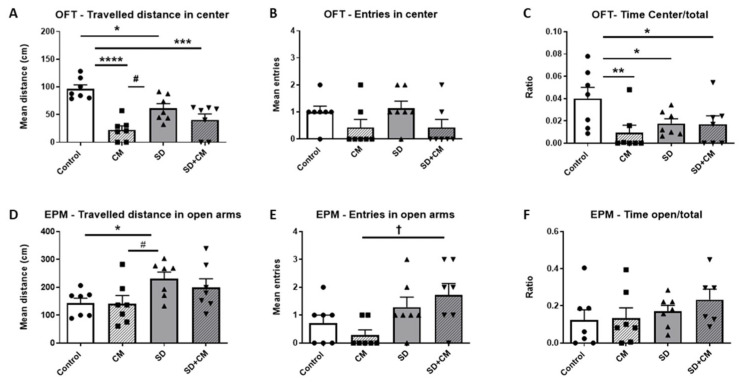
The effects *C. mas* administration on and emotionality-like behaviour in the Open Field Test (OFT) ((**A**)—distance travelled in centre, (**B**)—centre entries, (**C**)—ratio of time spent in centre/total) and the elevated plus maze (EPM) ((**D**)—distance travelled in open arms, (**E**)—open arms entries, (**F**)—ratio of time spent in open arms/total). Control—control group; CM—*C. mas* group; SD—sleep deprivation group; SD + CM—sleep deprivation with *C. mas* group; Results are expressed as mean ± SEM (n = 6–7). *, **, ***, **** indicate significant differences at *p* < 0.05, *p* < 0.01, *p* < 0.001, and *p* < 0.0001 when compared to the control group; # indicates a significant difference at *p* < 0.05 when compared to the SD group; † indicates a significant difference at *p* < 0.05 when compared to the CM group.

The effects of *C. mas* administration on emotionality-like behaviour in the OFT and EPM are illustrated in Figure 7. In regard to the OFT parameters, two-way ANOVA revealed a main effect of treatment in the case of distance travelled in centre (F = 29.75, η^2^ = 0.46, *p* < 0.0001), centre entries (F = 5.65, η^2^ = 0.19, *p* < 0.05), time spent in centre/total (F = 4.5, η^2^ = 0.13, *p* < 0.05), and a significant interaction effect between sleep and treatment in the case of distance travelled in centre (F = 9.52, η^2^ = 0.15, *p* < 0.01). In the case of the EPM parameters, two-way ANOVA revealed a main effect of sleep for distance travelled in open arms (F = 7.73, η^2^ = 0.24, *p* < 0.05) and number of entries in open arms (F = 9.48, η^2^ = 0.27, *p* < 0.01). No significant interaction effects were observed in the case of EPM parameters related to emotionality-like behaviour. Compared to control, the SD group travelled less in the centre of the arena (Figure 7A, *p* < 0.05), spent less time in the centre of the arena (Figure 7C, *p* < 0.05), and travelled more in the open arms (Figure 7D, *p* < 0.05). In addition, the SD group presented a higher number of entries into the open arms (Figure 7E, *p* > 0.05) and spent more time in the open arms (Figure 7F, *p* > 0.05) compared to the control group. The CM group travelled less in the centre of the arena (Figure 7A, *p* < 0.0001) and spent less time in the centre (Figure 7C, *p* < 0.01) compared to the control group. Furthermore, the CM group travelled less in the centre (Figure 7A, *p* < 0.05) and in the open arms (Figure 7D, *p* < 0.05) compared to the SD group.

### 3.3. Brain Histopathology

**Figure 8:**
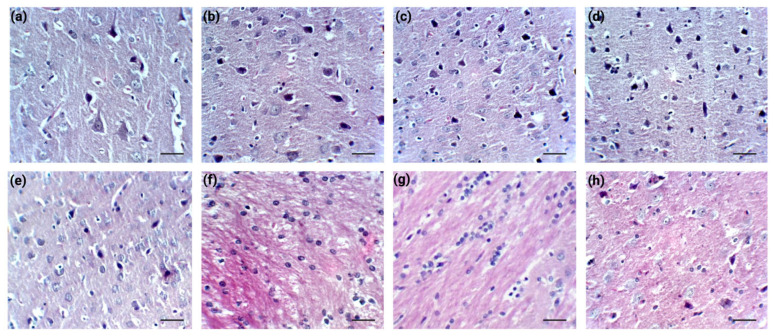
Effects of *C. mas* administration on the prefrontal cortex ((**a**–**d**); (**a**)—Control, (**b**)—CM, (**c**)—SD, (**d**)—SD + CM group) and corpus callosum myelin ((**e**–**h**); (**e**)—Control, (**f**)—CM, (**g**)—SD, (**h**)—SD + CM group). Haematoxylin–Eosin stain ×400. Scale bar = 25 µm.

Histological changes were evaluated in the prefrontal cortex and corpus callosum myelin through HE staining (n = 2/group), with representative images being presented in Figure 8 (Figure 8a–d prefrontal cortex; Figure 8e–h corpus callosum).

Cortical histological analyses showed relatively similar changes in the SD and CM groups (Figure 8b,c), with the appearance of dark neurons with mostly normal nuclear shape and occasional neuronal body atrophy and slight karyolysis processes (especially in the SD group). In animals previously exposed to SD and subsequently treated with *C. mas* (Figure 8d), mild neuronal atrophy, and the presence of dark neurons were observed, possibly indicative of endoplasmic reticulum stress.

In the corpus callosum, *C. mas* administration (Figure 8f) led to increased interstitial eosinophilic deposits, a higher oligodendrocyte count, and enhanced myelin eosinophilia. Similarly, more pronounced changes were observed in the SD group (Figure 8g), indicating possible tissue wear through potential disruptions in metabolic exchanges between the brain and blood. When the extract was administered during SD (Figure 8h), a prominent oligodendrocyte decrease was noted, along with the clearance of eosinophilic deposits from the tissue.

### 3.4. Brain Transmission Electron Microscopy (TEM) Ultrastructure Changes

**Figure 9:**
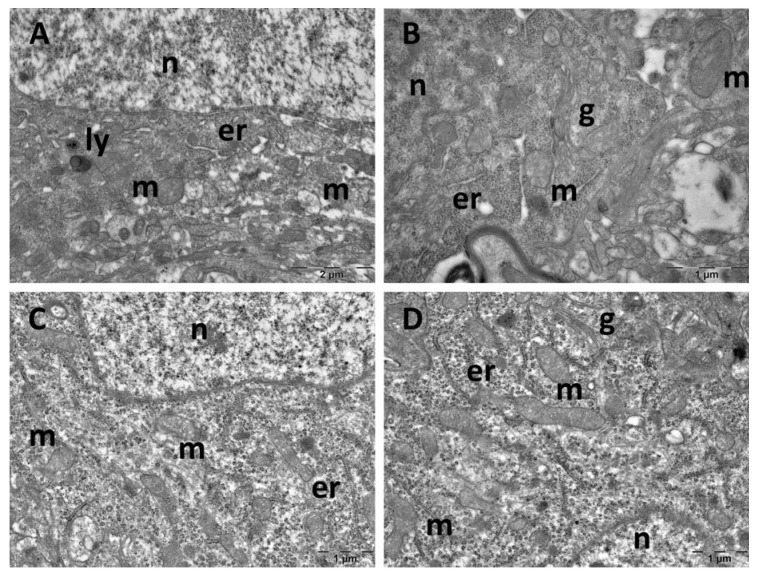
Ultrastructural aspects of the prefrontal cortex: (**A**,**B**): control group; (**C**,**D**): *C. mas* extract group. er: endoplasmic reticulum; g: Golgi complex; ly: lysosome; m: mitochondria; n: nucleus.

**Figure 10:**
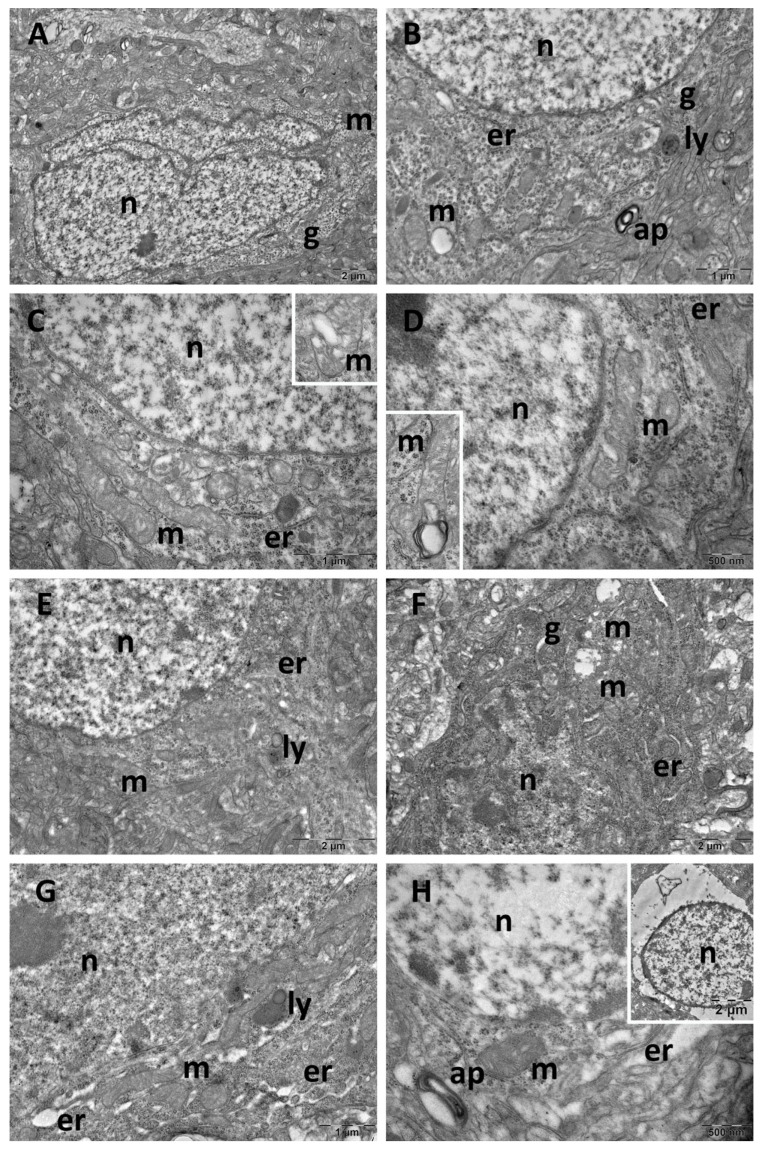
Ultrastructural aspects of the prefrontal cortex: (**A**–**D**): sleep deprivation group; (**E**–**H**): sleep deprivation+ *C. mas* extract group. ap: autophagosome; er: endoplasmic reticulum; g: Golgi complex; ly: lysosome; m: mitochondria; n: nucleus.

**Figure 11:**
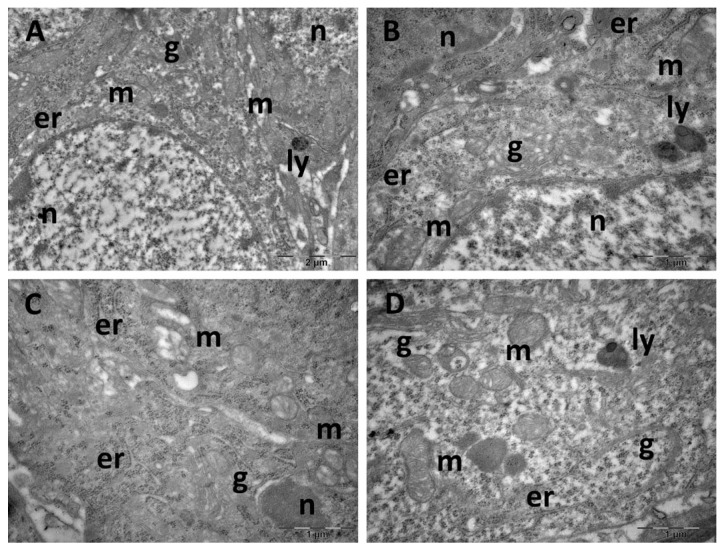
Ultrastructural aspects of the hippocampus: (**A**,**B**): control group; (**C**,**D**): *C. mas* extract group. er: endoplasmic reticulum; g: Golgi complex; ly: lysosome; m: mitochondria; n: nucleus.

**Figure 12:**
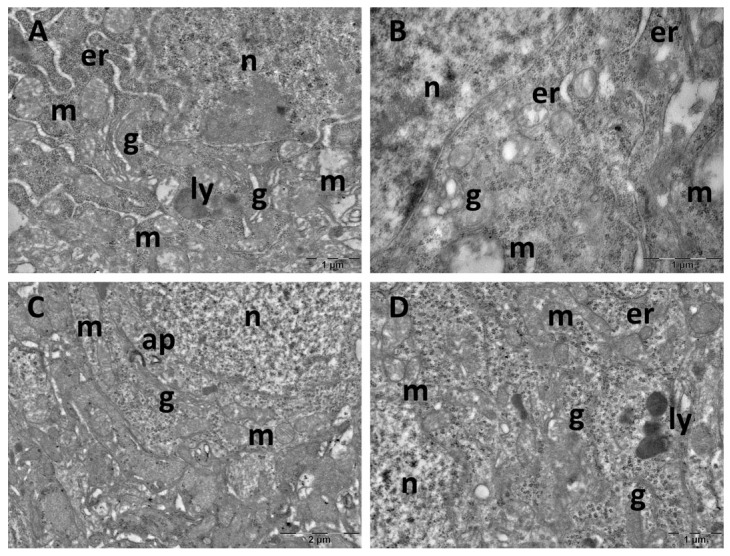
Ultrastructural aspects of the hippocampus: (**A**,**B**): sleep deprivation group; (**C**,**D**): sleep deprivation+ *C. mas* extract group. ap: autophagosome; er: endoplasmic reticulum; g: Golgi complex; ly: lysosome; m: mitochondria; n: nucleus.

**Figure 13:**
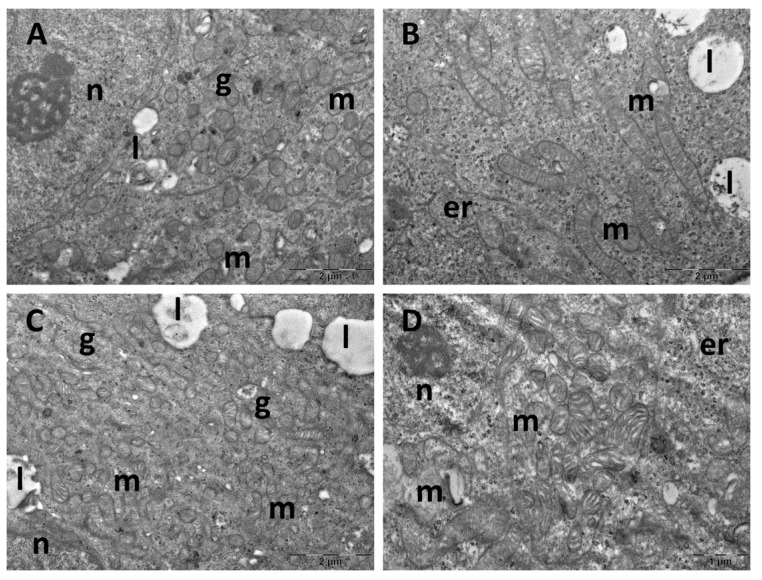
Ultrastructural aspects of the pineal gland: (**A**,**B**): control group; (**C**,**D**): *C. mas* extract group. er: endoplasmic reticulum; g: Golgi complex; l: lipid droplet; m: mitochondria; n: nucleus.

**Figure 14:**
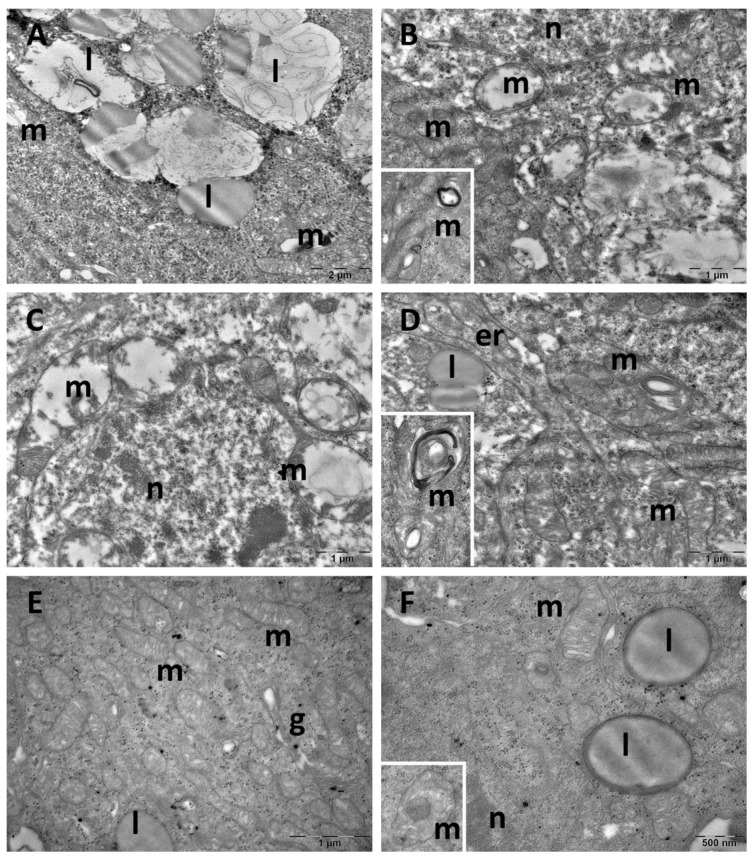
Ultrastructural aspects of the pineal gland: (**A**–**D**): sleep deprivation group; (**E**,**F**): sleep deprivation+ *C. mas* extract group. g: Golgi complex; l: lipid droplet; m: mitochondria; n: nucleus.

Ultrastructure changes were evaluated through TEM in the prefrontal cortex (Figure 9 and Figure 10), hippocampus (Figure 11 and Figure 12), and pineal gland (Figure 13 and Figure 14) (n = 2/group).

The neurons of the prefrontal cortex in the control group exhibited a normal ultrastructure. They had large, mostly euchromatic nuclei, with rounded shapes and regular contours. In the cytoplasm, free ribosomes and 2–3 Golgi complexes were present, alongside endoplasmic reticulum profiles and numerous, oval mitochondria with prominent cristae. Occasional lysosomes were also noted (Figure 9A,B). In the CM group, no ultrastructural alterations were observed, the aspect of the cells being comparable to that of the control group (Figure 9C,D). SD group neurons exhibited irregularly shaped nuclei, autophagosomes, and significant mitochondrial ultrastructural alterations (Figure 10A,B). These alterations included polymorphic mitochondria, vesicular cristae with an electron-lucent matrix, and mitochondria containing membrane whorls (Figure 10C,D). In the SD + CM extract group, some of the cells presented a normal ultrastructural aspect (Figure 10E). Many dark neurons were found, with irregularly shaped nuclei and electron-dense cytoplasm, where expanded profiles of endoplasmic reticulum and ballooned mitochondria with an electron-lucent matrix were noted (Figure 10F). Expanded endoplasmic reticulum profiles were also observed in normal neurons, along with occasional autophagosomes (Figure 10G,H). Additionally, a necrotic astrocyte was also present (Figure 10H).

In the hippocampus of the control group, neurons exhibited normal ultrastructure with rounded euchromatic nuclei, abundant free ribosomes, endoplasmic reticulum profiles, and Golgi complexes. Mitochondria were small and oval or rounded, with occasional lysosomes present (Figure 11A,B). While most cells from the CM group maintained a normal ultrastructure, some neurons group presented mitochondria with sparse cristae and an electron-lucent matrix (Figure 11C,D). In the SD group, most observed neurons were dark neurons, characterised by electron-dense cytoplasm and irregularly shaped nuclei (Figure 12A). In most cells, expanded endoplasmic reticulum profiles were noted, as well as ballooned mitochondria, with an electron-lucent matrix (Figure 12A,B). Neurons in the SD + CM group maintained a mostly normal ultrastructure, with occasional autophagosomes and irregularly shaped mitochondria (Figure 12C,D).

Control group pinealocytes exhibited a normal aspect with large nuclei, prominent nucleoli, abundant cytoplasm with Golgi complexes, endoplasmic reticulum profiles, and numerous free ribosomes. Lipid droplets were observed along with abundant round or oval mitochondria, that occasionally displayed irregular cristae (Figure 13A,B). Most of the cells in the CM group had a normal ultrastructure (Figure 13C). However, in some cells, mitochondria with electron-dense inclusions were noted (Figure 13D). In the SD group, large accumulation of homogenous and heterogenous lipid droplets were observed in several regions (Figure 14A). Some pinealocytes exhibited necrosis, with rarefied cytoplasm and ballooned mitochondria, characterised by an electron-lucent matrix and nearly absent cristae (Figure 14B,C). Other cells displayed mitochondria with vesicular cristae, membrane whorls, or electron-dense inclusions (Figure 14B,D). In the SD + CM group, pinealocytes maintained a mostly normal structure, with no observed necrotic cells (Figure 14E,F). However, some limited mitochondria contained paracrystalline, electron-dense inclusions (Figure 14F).

### 3.5. Oxidative Stress and Inflammatory Cytokine Changes

**Figure 15:**
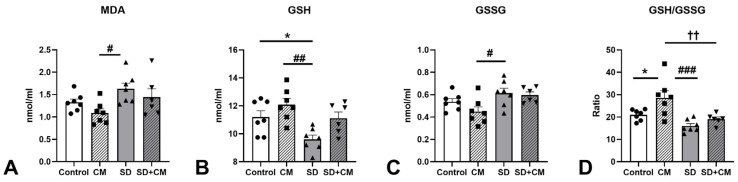
Effects of *C. mas* administration on Malondialdehyde (MDA) (**A**), oxidised Glutathione (GSH) (**B**), reduced Glutathione (GSSG) (**C**), and GSH/GSSG ratio (**D**) in serum; Control—control group; CM—*C. mas* group; SD—sleep deprivation group; SD + CM—sleep deprivation with *C. mas* group; Results are expressed as mean ± SEM (n = 6–7/group); * indicates a significant difference at *p* < 0.05 when compared to control; #, ##, ### indicate significant differences at *p* < 0.05 *p* < 0.01, and *p* < 0.001 when compared to SD; †† indicates a significant difference at *p* < 0.01 when compared to the CM group.

**Figure 16:**
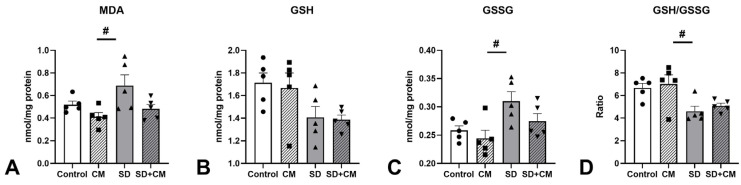
Effects of *C. mas* administration on Malondialdehyde (MDA) (**A**), oxidised Glutathione (GSH) (**B**), reduced Glutathione (GSSG) (**C**), GSH/GSSG ratio (**D**) in the prefrontal cortex; Control—control group; CM—*C. mas* group; SD—sleep deprivation group; SD + CM—sleep deprivation with *C. mas* group; Results are expressed as mean ± SEM (n = 5/group); # indicates a significant difference at *p* < 0.05 when compared to SD.

**Figure 17:**
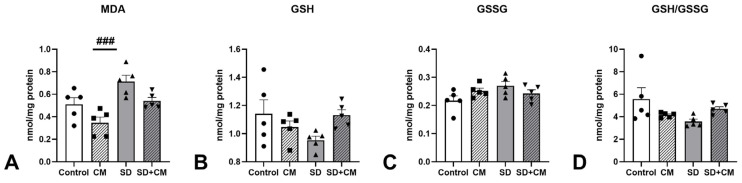
Effects of *C. mas* administration on Malondialdehyde (MDA) (**A**), oxidised Glutathione (GSH) (**B**), reduced Glutathione (GSSG) (**C**), GSH/GSSG ratio (**D**) in the Hippocampus; Control—control group; CM—*C. mas* group; SD—sleep deprivation group; SD + CM—sleep deprivation with *C. mas* group; Results are expressed as mean ± SEM (n = 5/group); ### indicates a significant difference at *p* < 0.001 when compared to SD.

Multiple OS parameters (MDA, GSH, GSSG, GSH/GSSG ratio) were evaluated in the prefrontal cortex, hippocampus, and serum. The results are presented in Figure 15Figure 16 and Figure 17.

In the serum, two-way ANOVA revealed a main effect of sleep in the case of MDA (F = 7.29, η^2^ = 0.22, *p* < 0.05) and GSSG (F = 8.94, η^2^ = 0.25, *p* < 0.01). A main effect of treatment and sleep was observed in the case of GSH (F = 8.50, η^2^ = 0.20, *p* < 0.01 and F = 9.69, η^2^ = 0.23, *p* < 0.01) and GSH/GSSG ratio (F = 8.23, η^2^ = 0.17, *p* < 0.01 and F = 15.58, η^2^ = 0.32, *p* < 0.001). No significant interaction effect between treatment and sleep was observed. Compared to the control group, SD resulted in increased levels of MDA and GSSG, along with decreases in GSH and the GSH/GSSG ratio. Serum GSH was significantly decreased in the SD group when compared to control (11.2 ± 1.20 vs. 9.60 ± 0.813 nmol/mL, *p* < 0.05), suggesting elevated serum OS. Compared to the CM group, SD determined significant increases in MDA and GSSG coupled with significant reductions in GSH and GSH/GSSG ratio (MDA, *p* < 0.05; GSSG, *p* < 0.05; GSH, *p* < 0.01; GSH/GSSG ratio, *p* < 0.001), suggesting a potential protective effect of the *C. mas* extract on the examined OS parameters. Compared to SD alone, treatment with *C. mas* in the SD + CM group revealed a nonsignificant tendency towards normalising the examined OS parameters (MDA 1.63 ± 0.333 vs. 1.44 ± 0.456; GSH 9.60 ± 0.813 vs. 11.1 ± 1.10; GSSG 0.614 ± 0.116 vs. 0.596 ± 0.068; GSH/GSSG ratio 16.0 ± 2.78 vs. 19.0 ± 2.36 nmol/mL; all *p* > 0.05).

In the prefrontal cortex, two-way ANOVA revealed a significant main effect of treatment in the case of MDA (F = 7.41, η^2^ = 0.26, *p* < 0.05) and a significant main effect of sleep in case of GSH (F = 9.36, η^2^ = 0.37, *p* < 0.01), GSSG (F = 9.20, η^2^ = 0.32, *p* < 0.01), and GSH/GSSG ratio (F = 14.58, η^2^ = 0.47, *p* < 0.01). No significant interaction effect between treatment and sleep was observed. Similar to serum, compared to the control group, SD determined nonsignificant increases in MDA and GSSG coupled with decreases in GSH and GSH/GSSG ratio (GSH 1.71 ± 0.197 vs. 1.41 ± 0.217, GSSG 0.258 ± 0.017 vs. 0.310 ± 0.037, GSH/GSSG 6.66 ± 0.916 vs. 4.60 ± 1.02, MDA 0.519 ± 0.069 vs. 0.687 ± 0.215 nmol/mg prot., all *p* > 0.05). Compared to the CM group, SD resulted in significant reduction in the GSH/GSSG ratio coupled with significant increases in MDA and GSSG values (*p* < 0.05), suggesting a potential neuroprotective effect of the cornelian cherry fruit extract. As in the case of serum, treatment with *C. mas* in the SD + CM group revealed a potential modulatory action on the effects of SD through a nonsignificant tendency towards normalising mainly MDA and GSSG values (MDA 0.687 ± 0.215 vs. 0.481 ± 0.090; GSSG 0.310 ± 0.037 vs. 0.275 ± 0.030 nmol/mg prot., all *p* > 0.05).

In the hippocampus, two-way ANOVA revealed a main effect of treatment and sleep in the case of MDA (F = 10.89, η^2^ = 0.26, *p* < 0.01 and F = 15.19, η^2^ = 0.36, *p* < 0.01). A significant interaction effect was observed in the case of GSH (F = 5.24, η^2^ = 0.23, *p* < 0.05), GSSG (F = 4.72, η^2^ = 0.20, *p* < 0.05) and GSH/GSSG ratio (F = 5.66, η^2^ = 0.24, *p* < 0.05). No significant interaction effect between treatment and sleep was observed. SD determined increases in MDA and GSSG values coupled with decreases in GSH and GSH/GSSG compared to control (MDA 0.510 ± 0.134 vs. 0.712 ± 0.126, GSH 1.14 ± 0.222 vs. 0.953 ± 0.065, GSSG 0.217 ± 0.038 vs. 0.270 ± 0.035, GSH/GSSG 5.56 ± 2.27 vs. 3.57 ± 0.469 nmol/mg prot.) without reaching statistical significance (all *p* > 0.05). Compared to the CM group, SD determined significant increases in hippocampal MDA (0.346 ± 0.114 vs. 0.712 ± 0.126, *p* < 0.001) coupled with nonsignificant decreases in GSH, GSH/GSSG ratio (*p* > 0.05), and increases in GSSG (*p* > 0.05), suggesting a potential neuroprotective effect on hippocampal lipid peroxidation. As in the case of serum and cortex samples, treatment with *C. mas* in the SD + CM group demonstrated a nonsignificant trend towards normalisation of the examined parameters (MDA 0.712 ± 0.126 vs. 0.540 ± 0.069; GSH 0.953 ± 0.065 vs. 1.13 ± 0.087, GSSG 0.270 ± 0.035 vs. 0.243 ± 0.029, GSH/GSSG 3.57 ± 0.469 vs. 4.69 ± 0.441 nmol/mg prot.; all *p* > 0.05).

**Figure 18:**
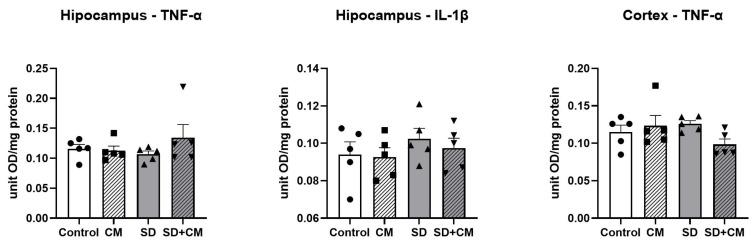
Effects of *C. mas* administration on oxidised Tumour Necrosis Factor Alpha (TNF-α) and Interleukin-1 beta (IL-1β) in the prefrontal cortex and hippocampus of rats; Control—control group; CM—*C. mas* group; SD—sleep deprivation group; SD + CM—sleep deprivation with *C. mas* group; Results are expressed as mean ± SEM (n = 5/group).

Inflammatory cytokines were evaluated in the prefrontal cortex (TNF-α) and hippocampus (TNF-α, IL-1β). The results are presented in Figure 18. Two-way ANOVA revealed no significant effects of treatment, sleep, or interaction between the groups in regard to the examined parameters (all *p* > 0.05).

### 3.6. Serum BDNF, GABA and Corticosterone

**Figure 19:**
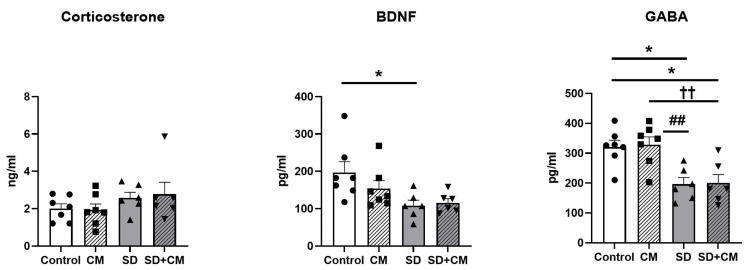
Effects of *Cornus mas* administration on serum BDNF, GABA, and Corticosterone; Control—control group; CM—*C. mas* group; SD—sleep deprivation group; SD + CM—sleep deprivation with *C. mas* group; Results are expressed as mean ± SEM (n = 6–7/group); * indicates a significant difference at *p* < 0.05 when compared to control; ## indicates a significant difference at *p* < 0.01 when compared to SD; †† indicates a significant difference at *p* < 0.01 when compared to CM.

BDNF, GABA and Corticosterone were evaluated through ELISA in serum samples. The results are presented in Figure 19. In the case of serum CORT, two-way ANOVA revealed no significant differences between the groups (*p* > 0.05), suggesting a lack of a stress response in the animals exposed to curtailed sleep. Two-way ANOVA revealed a significant main effect of sleep in the case of BDNF and GABA (F = 9.03, η^2^ = 0.27, *p* < 0.01 and F = 25.27, η^2^ = 0.53, *p* < 0.0001). Compared to control, the SD group presented with significantly decreased BDNF and GABA values (*p* < 0.05). In addition, compared to the CM group, SD determined a significant decrease in serum GABA levels (*p* < 0.01).

## 4. Discussion

The results obtained in this study indicate that seven days of prolonged PSD may induce behavioural changes in rodents, accompanied by systemic OS through the depletion of GSH, decreased serum BDNF, GABA and morphological as well as ultrastructural alterations in several brain regions. While *C. mas* showed effectiveness in modulating certain features of the SD-induced behavioural phenotype, its effects on other associated alterations were markedly limited and primarily detectable at the ultrastructural level.

In this study, we utilised a cornelian cherry extract derived from a local serovar cultivated in Cluj County, Romania (temperate climate). While our analysis revealed only one anthocyanin (cyanidin-3-glucoside) in the *C. mas* extract, other studies identified further anthocyanins in various *C. mas* extracts such as delphinidin 3-galactosid, cyanidin-3-O-galactosid, and pelargonidin [51]. The phytochemical content of *C. mas* fruits is known to vary significantly due to a range of factors, including the plant variety, serovar, environmental conditions, and climate [52]. Mechanistically, *C. mas* may exert its antioxidant effects by reducing oxidative damage and enhancing key antioxidant systems such as the GSH/GSSG ratio and enzymes like CAT, PON1, and GPx [26,28,29].

PSD of varying durations has been consistently shown to induce a mania-like phenotype in both mice and rats [53,54,55,56,57], most notably characterised by hyperactivity [54,57]. In this study, seven days of PSD induced a behavioural phenotype characterised by hyperlocomotion and hyperactivity, which were modulated by *C. mas* administration. This study revealed conflicting OFT and EPM findings in regard to anxiety-like behaviour. Previous studies have suggested that the mania-like phenotype frequently observed in rodent SD models may mask anxiety-related behavioural alterations [58]. While SD seems to reliably induce anxiety in humans [59], preclinical data are less consistent [58]. In rodents, anxiety may manifest as hyperactivity, potentially serving an adaptive function in response to aversive stimuli such as SD [60].

TEM analysis in this study revealed minimal ultrastructural alterations in the CM group, while significant changes (particularly in mitochondrial morphology) were observed in the SD group within the prefrontal cortex, hippocampus, and pineal gland. Furthermore, these changes were mostly normalised in the SD + CM group, suggesting a potential protective effect of the fruit extract. Both PSD and TSD protocols have been shown to alter hippocampal ultrastructure in rats, including mitochondrial changes [53,61,62,63], microglial activation [53], myelin disruption [53], blood–brain barrier alterations [53], neuronal and glial apoptosis [53,63], synaptic abnormalities [64], and cellular debris accumulation [65]. Ultrastructural changes in rodent cortical regions appear less pronounced than in the hippocampus. Observed alterations include altered ER morphology and increased organelle contact points (motor cortex) [66], modest mitochondrial and lysosomal changes (frontal cortex layer II) [67], and progressive neuronal and synaptic degeneration with prolonged SD (anterior limbic cortex) [68]. Mechanistically, PSD may induce a stressful microenvironment through OS and inflammation, which triggers neuronal damage, demyelination, and vascular disruption, facilitating peripheral immune infiltration and further amplifying neuroinflammation and degeneration [53].

Our findings suggest that *C. mas* administration and PSD may contribute to myelin disruption, as indicated by structural changes in the corpus callosum histology, which align with previous rodent studies on PSD and TSD in the cortex and corpus callosum [53,69]. Recent MRI imagining data seem to further highlight that brain white matter may be particularly sensitive to acute SD in humans [70]. Notably, disruptions in oligodendrocyte cholesterol homeostasis have been suggested as a potential mechanism underlying impaired myelin function during SD (preprint [71]).

Compared to other CNS sites, reports on the pineal gland ultrastructure following SD are scarce. In this study, we showed that SD induces several changes in pinealocytes suggestive of increased cell activity including lipid droplet accumulation, mitochondrial alterations, and even necrosis—findings relatively consistent with previous reports of TSD-induced ultrastructural damage [72] or constant exposure to darkness [73]. Pineal gland alterations and melatonin disruption are closely linked to damage in other brain sites. Prolonged TSD in weanling rats has been shown to reduce melatonin synthesis [74], potentially inducing OS and neuronal damage in other brain sites such as the hippocampus [75]. Compared to the SD group, cells in the SD + CM group largely preserved their normal structure, suggesting a potential protective effect of the *C. mas* extract.

One proposed function of sleep is redox balance homeostasis [76]. The majority of evidence seems to point to a consistent increase in OS markers in both serum [11] and various brain regions (e.g., whole brain, hippocampus, cortex, thalamus, hypothalamus, amygdala, and locus coeruleus) in rodent models of PSD or TSD [11,12]. Specifically, we previously showed that most data points in the serum (11/13, 84.61%), hippocampus (20/24, 83.33%), and cortex (5/7, 71.42%) revealed changes suggestive of altered redox balance [11]. However, it should be noted that extensive variability in SD protocols, animal strains, and examined parameters may partly account for differences in results [11,12].

Consistent with previous findings, this study observed a decrease in GSH levels and the GSH/GSSG ratio, alongside increased GSSG and MDA, in the SD group compared to the control group across the examined body sites. These changes were biologically plausible and consistent across serum, prefrontal cortex, and hippocampus, with statistical significance reached only for serum GSH, indicating systemic OS through the depletion of GSH. GSH is considered the most abundant intracellular antioxidant and the first line of defence against OS (reviewed in [77]). Cellular damage by reactive oxygen species (ROS) is prevented through the detoxification of peroxides (e.g., H_2_O_2_) by Glutathione peroxidase (GPx) using GSH as an electron donor, resulting in GSSG as the end product. Further reduction in GSSG is performed through GSH reductase (GR) in a NADPH-dependent process (reviewed in [78]). While MDA changes did not reach statistical significance, low level damage to membrane lipids cannot be excluded based on our data. Additionally, lipid degradation in the context of energy production through autophagy has been previously proposed to explain low levels of lipid peroxidation during SD in rodents [79]. The significant differences observed between the CM and SD groups across the examined sites suggest a potential protective effect of the *C. mas* extract. However, this potential effect requires further in-depth confirmation in future studies.

Redox and inflammatory pathways are highly interconnected, linked through various bidirectional molecular signalling cascades governed by transcription factors such as NF-κB and Nrf2 (reviewed in [80,81]). Furthermore, there is considerable crosstalk between the immune system and sleep, as evidenced through the essential roles of cytokines such as IL-1β and TNF-α (as well as others) in sleep regulation (reviewed in [82]). Multiple lines of evidence seem to support the fact that SD might determine an inflammatory response (reviewed in [82]). However, it is worth noting that both rodent [53,83,84] and human studies [82,85] currently offer mixed results in regard to changes in cytokine levels following SD. Mechanistically, SD has been shown to induce cognitive dysfunction in rats, explained through blood–brain barrier dysfunction and neuroinflammation (IL-1β, IL-6, TNFα, iNOS, COX2) due to the activation of the TLR4/MyD88/NF-κB pathway [84]. Furthermore, prolonged and not acute SD may be required in order to elicit an inflammatory response [86]. Unlike the OS parameters, inflammatory cytokine levels in cortical and hippocampal samples did not show significant differences in our study. These results are in accordance with previously outlined data [86]. Although downstream inflammatory factors such as cytokines may not be elevated during SD, upstream signalling factors (e.g., NF-κB, IL-6 and TNF mRNA) may serve as more sensitive indicators and have been shown to exhibit an increase even after short-term SD in humans [87].

Compared to the control, the CM group exhibited several decreased behavioural parameters, coupled with histological changes and a significantly increased serum GSH/GSSG ratio. However, these changes were not consistent across the examined parameters and may only potentially point towards a modest sedative effect of the extract. While *C. mas* has not been traditionally used for enhancing sleep duration or quality, recent research suggests that cornelian cherry fruits contain higher doses of melatonin compared to other fruits (e.g., sour and sweet cherries) [88] that are known in clinical trials to improve both mood and several sleep parameters [89,90,91]. Interestingly, beyond its established roles in circadian regulation and antioxidative defence, exogenous melatonin also appears to modulate locomotor activity in rodents. In rats, i.p administration of higher but not lower doses of exogenous melatonin has been associated with a reduction in spontaneous locomotor activity [92]. Previous studies have indicated that the administration of relatively high doses of antioxidants (mainly flavonoids) in rodents may exert direct or indirect suppressive effects on locomotor activity in various behavioural tests (e.g., 5-methoxyflavone [93], tangeretin [94], nerolidol [95], apigenin, safranal, among others—reviewed in [96], alpha-pinene [97]). Furthermore, several flavonoid glycosides such as hersperidin, diosmin, naringin, gossipyn, and rutin have shown a CNS depressant effect and decreased locomotor activity in rodents following i.p administration [98]. Among these, *C. mas* is known as a potentially valuable source of quercetin 3-O-rutinoside (rutin) [99]; thus, at least partially explaining our results. Loganin can be considered a major iridoid found in both *Cornus officinalis* and *Cornus mas* [99]. Interestingly, loganin has been reported to exert inhibitory effects on spontaneous locomotor activity in rodents, along with sedative and hypnotic properties [100,101], explained through an interaction with GABAergic neurons and the serotonergic system [101]. Several further potential mechanisms have been outlined for the observed effects of the previously mentioned compounds such as a sedative effect through the interaction with adenosine, GABA_A_, glycine, NDMA receptors in the case of 5-methoxyflavone [93], or modulation of GABA_A_ receptors in the case of tangeretin [94]. While yet mostly unexplored, several other compounds from *C. mas* might also determine similar effects on locomotor activity in rodents. Maintaining normal ROS levels is essential for brain functioning. ROS are known to exhibit essential physiological roles, such as cellular signalling (reviewed in [102]). Interestingly, overexpression of endogenous antioxidant systems has been shown to influence behaviour in fruit flies (e.g., sleep behaviour) [10]; furthermore, overexpression of CAT in rodent astrocytes has been shown to decrease mitochondrial ROS and induce both metabolic and behavioural alterations (e.g., decrease in locomotor and exploratory behaviour) [103]. While unlikely, it could be the case that higher doses of exogenous antioxidants could potentially play a similar role in the suppression of physiological ROS required for brain function and exploratory behaviour. In this study, the CM group showed no significant differences in oxidative parameters compared to the control group, except for serum GSH/GSSG levels. Although limited, this finding may suggest a mechanism involving excessive antioxidant activity, as previously discussed.

In accordance to our results, previous studies have shown reductions in serum levels of various neurotransmitters such as BDNF and GABA following prolonged SD protocols in rodents [104,105]. BDNF is vital for sleep, neuroplasticity, and memory. While short SD might elevate circulating BDNF and provide a rapid antidepressant effect, prolonged SD seems to determine an increased stress vulnerability and a subsequent decrease in BDNF levels (reviewed in [106]). Reduced circulating BDNF levels have been reported in individuals with bipolar disorder during both manic and depressive episodes, showing a negative correlation with symptom severity [107], and have also been observed in patients with insomnia [108]. Similarly, reduced circulating GABA levels have been observed during depressive episodes in patients with BD [109]. Mechanistically, prolonged SD may lead to TrkB receptor (Tropomyosin receptor kinase B) overstimulation through sustained hyperarousal and stress-related pathways, ultimately leading to diminished signalling, receptor desensitisation, and subsequent decreased central and peripheral levels of BDNF (reviewed in [108]).

Stress can be considered a significant confounding factor in rodent SD studies and most available protocols seem to be stress-inducing to various degrees (reviewed in [110]). The absence of significant increases in serum CORT in the SD group align with previous studies and simultaneously contrast others [11], possibly suggesting that the observed alterations were more likely a direct consequence of SD rather than a secondary effect of stress.

To the best of our knowledge, this is the first experimental study aimed at exploring the behavioural and neuroprotective potential of a cornelian cherry extract (*C. mas*) in the context of SD. However, this study has several limitations. It was conducted on adult male Wistar rats through PSD, limiting generalisability across animal strains, sexes, ages [11,12], and most likely different SD protocols. Additionally, upstream regulators like NF-κB and Nrf2 were not evaluated in this study and may be more sensitive indicators compared to downstream elements. A further limitation of the present study is the limited sample size, particularly in the case of the behavioural assessments, histological examinations, and ultrastructural evaluations. Clinical translation remains limited. To the best of our knowledge, to date, no clinical trials have evaluated the neuroprotective effects of natural compounds during SD in humans.

The modest effects of the *C. mas* extract could be attributed to several factors including reduced bioavailability of the major active compounds through oral administration (e.g., polyphenols and specifically flavonoids in both humans and animals [111,112], iridoids such as loganic acid in rats [113]), bioactive compound variability determined by *C. mas* cultivar and growing conditions [16,18], type of extract used [18], and short-term administration duration combined with a prolonged SD protocol without sleep recovery intervals. Furthermore, although *C. mas* has previously shown some neuroprotective effects in rodents [30,31], it is possible that certain bioactive compounds may exhibit reduced or even absent permeability across the blood–brain barrier [114].

## 5. Conclusions

Taken together, our findings suggest that prolonged sleep deprivation in rats induces behavioural changes characterised by hyperactivity and hyperlocomotion. Morphologically, these changes are potentially linked to histological and ultrastructural alterations in several brain regions such as the prefrontal cortex, myelin, hippocampus, and pineal gland. Furthermore, sleep deprivation determined systemic oxidative stress, characterised by GSH depletion, coupled with reductions in circulating BDNF and GABA levels. However, we observed no changes regarding brain inflammatory cytokines TNF-α and IL-1β in the prefrontal cortex and hippocampus.

The effects of *C. mas* fruit extract were limited across most examined parameters. However, a modest modulatory activity was noted in behavioural measures and ultrastructural changes. This activity can potentially be explained through its antioxidant effects. Further studies are warranted to clarify the underlying modulatory potential, underlying mechanisms, and to evaluate different extract types, dosages, and at different durations of sleep deprivation.

## Figures and Tables

**Table 1:** Total polyphenolic content and DPPH antioxidant activity (mean value ± SD, n = 3).

	Total Polyphenol Content (g GAE/1000 mL)	Antioxidant Activity—DPPH (mg TE/mL)
*C. mas* fruit extract	25.5 ± 0.6639	4.8497 ± 0.0094

GAE—Gallic acid equivalents; TE—Trollox equivalent; DPPH—2,2-diphenyl-1-picrylhydrazyl assay.

**Table 2:** *Cornus mas*—Identification and quantification of bioactive compounds using LC-MS (mean value ± SD, n = 3).

Chemical Class		Individual Compounds	Concentration (µg/mL)
**Phenolic acids**	Hydroxycinnamic acids	Caftaric acid	35.992 ± 0.71984
		Gentisic acid	2.922 ± 0.35064
		Caffeic acid	70.998 ± 7.80978
		Chlorogenic acid	70.647 ± 9.18411
	Hydroxybenzoic acids	Gallic acid	49.49 ± 6.4337
		Protocatechuic acid	13.97 ± 1.5367
**Flavonoids**	Flavonols	Quercetin	<LOQ *
		Kaempferol	<LOQ *
	Flavonol glycosides	Hyperoside	3.6 ± 0.324
		Isoquercitrin	37.476 ± 1.12428
		Rutoside	4.74 ± 0.0948
		Quercitrin	2.315 ± 0.20835
	Flavan-3-ols (catechins)	Epicatechin	0.62 ± 0.0372
		Catechin	0.05 ± 0.003
		Epigallocatechin	0.58 ± 0.0696
		Epigallocatechin gallate	1.08 ± 0.0648
**Anthocyanins**	Cyanidins	Cyanidin 3-glucoside	1162.241 ± 34.867

* <LOQ—below the limit of quantification for the analytical method; LC-MS—Liquid chromatography tandem mass spectrometry.

## Data Availability

Data are included in the Manuscript and further available from the corresponding author upon reasonable request.
